# Cellular Interleukin Enhancer-Binding Factor 2, ILF2, Inhibits Japanese Encephalitis Virus Replication In Vitro

**DOI:** 10.3390/v11060559

**Published:** 2019-06-17

**Authors:** Xiaofang Cui, Ping Qian, Tingting Rao, Yanming Wei, Fang Zhao, Huawei Zhang, Huanchun Chen, Xiangmin Li

**Affiliations:** 1 State Key Laboratory of Agricultural Microbiology, Huazhong Agricultural University, Wuhan 430070, China; cuixiaofang@webmail.hzau.edu.cn (X.C.); raoting@126.com (T.R.); YanmingWei@mail.hzau.edu.cn (Y.W.); zhaofang@webmail.hzau.edu.cn (F.Z.); azhangzhizhong@126.com (H.Z.); chenhch@mail.hzau.edu.cn (H.C.); 2 Laboratory of Animal Virology, College of Veterinary Medicine, Huazhong Agricultural University, Wuhan 430070, China; 3 Key Laboratory of Development of Veterinary Diagnostic Products, Ministry of Agriculture, Wuhan 430070, China; 4 Key Laboratory of Preventive Veterinary Medicine in Hubei Province, The Cooperative Innovation Center for Sustainable Pig Production, Wuhan 430070, China

**Keywords:** Japanese encephalitis virus, cellular interleukin enhancer-binding factor 2 (ILF2), antiviral activity, viral RNA translation

## Abstract

Japanese encephalitis virus (JEV) is a zoonotic mosquito-borne flavivirus which is the leading causative agent of viral encephalitis in endemic regions. JEV NS3 is a component of the viral replicase complex and is a multifunctional protein. In this study, interleukin enhancer-binding factor 2 (ILF2) is identified as a novel cellular protein interacting with NS3 through co-immunoprecipitation assay and LC-MS/MS. The expression of ILF2 is decreased in JEV-infected human embryonic kidney (293T) cells. The knockdown of endogenous ILF2 by special short hairpin RNA (shRNA) positively regulates JEV propagation, whereas the overexpression of ILF2 results in a significantly reduced JEV genome synthesis. Further analysis revealed that the knockdown of ILF2 positively regulates viral replication by JEV replicon system studies. These results suggest that ILF2 may act as a potential antiviral agent against JEV infection.

## 1. Introduction

Japanese encephalitis virus (JEV) belongs to the genus *Flavivirus* of the family Flaviviridae, which includes several critically important human pathogens such as West Nile virus (WNV), Dengue virus (DENV), and Zika virus [1,2,3]. The genome of JEV is a single-stranded and plus-sense RNA of approximately 11 kb in length. The genomic RNA carries the open reading frame (ORF) and encodes polyprotein cleaved by host and viral proteases to produce three structural proteins—capsid (C), pre-membrane (PrM), and envelope (E)—and seven nonstructural (NS) proteins—NS1, NS2A, NS2B, NS3, NS4A, NS4B, and NS5 [3]. The nonstructural proteins play an important role in viral replication, pathogenesis, and virus–host interactions. JEV NS3 is a multifunctional protein possessing helicase, protease, and nucleoside triphosphatase activities [4,5,6]. In addition, the 3’ NCR of JEV genomic RNA may form a replication complex together with NS3 and NS5 and participates in viral RNA replication [7,8].

JEV is recognized as the most significant cause of mosquito-borne viral encephalitis and a serious public health problem in Asia and the Pacific region, with over 3 billion persons at risk of infection [9,10,11,12]. The mortality is 20%–30% only among severe Japanese encephalitis (JE) cases; about 50% of patients develop neuropsychiatric or physiological sequelae. In spite of effective vaccine availability, there still are 68,000 reported cases of JEV annually [13,14,15]. Therefore, it is necessary to develop new strategies against JEV, especially broad-spectrum antiviral agents and small molecules that inhibit a critical step in the viral cycle [16,17].

Interleukin enhancer-binding factor 2 (ILF2), also known as nuclear factor 45 (NF45) in humans and mice, is a chromatin-interacting protein that is constitutively expressed and localized in the nucleus. ILF2 forms a stable heterodimer with interleukin enhancer-binding factor 3 (ILF3, also called NF90) and regulates the transcription of the *IL-2* gene during T cell activation in the nucleus [18]. ILF2 contains an N-terminal arginine- and glycine-rich (RGG) domain, and a domain associated with zinc fingers (DZF) homologous to ILF3. ILF2 and the ILF2/ILF3 complex have been indicated to act as important regulatory factors involved in microRNA biogenesis, RNA transcription, splicing, mRNA translation, DNA break repair, cell proliferation, and apoptosis [19,20,21,22,23,24,25,26]. Recent studies revealed ILF2 to be considered as an important host cellular protein that is involved in the replication process of viruses, including hepatitis B virus, bovine viral diarrhea virus (BVDV), hepatitis C virus, infectious bursal disease virus (IBDV), human immunodeficiency virus type 1 (HIV-1), as well as porcine reproductive and respiratory syndrome virus (PRRSV) [27,28,29,30,31,32,33]. Meanwhile, ILF2 represses the ATP-induced activation of endogenous NLRP3 inflammasome in macrophages [34].

JEV NS3 is a multifunctional protein that participates in viral RNA replication. In this study, we aim to identify novel host factors interacting with JEV NS3 and evaluate their potential role in the infection of JEV. To this end, 293T cells were transduced with recombinant lentivirus expressing JEV NS3 and liquid chromatography-tandem mass spectrometry (LC-MS/MS) analysis was performed. ILF2 was shown to interact with JEV NS3. Moreover, ILF2 was found to be involved in the JEV life cycle and inhibit JEV replication.

## 2. Materials and Methods

### 2.1. Cells and Viruses

Human embryonic kidney cells (293T) and baby hamster kidney BHK-21 cells (BHK-21) were grown in Dulbecco’s modified essential medium (DMEM; Invitrogen, USA) containing 10% fetal bovine serum (FBS; Gibco, NY, USA, 100 U/mL penicillin, and 10 μg/mL streptomycin sulfate at 37 °C in a humidified 5% CO_2_ incubator. JEV strain RP-9, which was a mutant of JEV Taiwanese isolate NT109 strain using irradiation, was kindly provided by Professor Yi-ling Lin (Institute of Biomedical Sciences, Academia Sinica, Taibei, Taiwan), and the titer assay was determined in BHK-21 cells [35].

### 2.2. Antibodies

Mouse monoclonal antibodies against Flag-tag and HA-tag were purchased from Medical and Biological Laboratories Co., Ltd. (MBL, Tokyo, Japan). Rabbit anti-JEV NS3 polyclonal antibodies and EasyBlot mouse anti IgG (HRP) were obtained from GeneTex Inc (USA). Rabbit anti-ILF2 polyclonal antibodies, rabbit anti-Lamin A/C polyclonal antibodies, mouse anti-alpha tubulin monoclonal antibodies, and mouse anti-glyceraldehyde-3-phosphate dehydrogenase (GAPDH) monoclonal antibodies were purchased from Proteintech Group Inc. (China). Horseradish peroxidase-conjugated (HRP) goat anti-mouse and goat anti-rabbit IgG (BA1054) (H + L) secondary antibodies were obtained from Boster Bioengineering Ltd. (China). Mouse Anti-JEV NS3 monoclonal antibodies (mAb) were kindly provided by Professor Sheng-bo Cao (Huazhong Agricultural University).

### 2.3. Plasmids

All primers used in this study are showed in Table 1. Expression plasmid for wild-type human ILF2 was constructed by polymerase chain reaction (PCR) amplification of cDNA from 293T cells. The *ILF2* gene was cloned into pCAGGS-HA and pTRIP-3Flag-REP vector to generate HA and Flag-tagged expression plasmids pCAGGS-HA-ILF2 and pTRIP-3Flag-ILF2. JEV NS3 was also cloned into pTRIP-3Flag-REP to generate pTRIP-3Flag-NS3. All recombinant plasmids were further verified by DNA sequencing.

pLKO.1 was purchased from Miaoling Bioscience and Technology Co., Ltd. (Wuhan, China) and used as a negative control, exhibiting no downregulation of any human gene. pLKO.1-shILF2, containing the short hairpin RNA (shRNA) corresponding to the ILF2 mRNA sequences (5’-CCAGCACCTGATGAAACTT-3’) (shILF2), was constructed and used to inhibit endogenous ILF2 protein expression. The plasmid carrying the JEV sub-genomic replicon fused with a Luciferase reporter with T7 promoter was kindly provided by Professor Bo Zhang (Wuhan Institute of Virology, Chinese Academy of Sciences). The plasmid carrying the JEV replication-deficient replicon with CMV promoter (a GDD-AAG mutation in NS5 of JEV replicon DNA) [36], pCR3.1-3Flag-NS1/NS5/E, and pmirGLO plasmids was kindly provided by Professor Yi-ling Lin. The firefly luciferase gene was amplified from pmirGLO and cloned into pCAGGS-HA.

### 2.4. Lentivirus Preparation

Lentiviral vectors pTRIP-3Flag-NS3, pTRIP-3Flag-ILF2, or pLKO.1-shILF2 were co-transfected into 293T cells with two helper plasmids, pCMV-VSVG and pCMV-Gag-po1, by using Lipofectamine 2000 (Invitrogen, California, USA) according to the manufacturer’s instructions. The supernatants were harvested and centrifuged at 5000 rpm/min for 10 min at 4 °C to discard cell debris, and then stored at −80 °C. Then, 293T cells were transduced with the recombinant lentiviruses in the presence of 6.0 µg/mL of polybrene (Sigma, Missouri, USA).

### 2.5. MTS Assay

At 36 h post-transduction of recombinant lentivirus carrying ILF2 or sh-ILF2, cells were seeded in 96-well plates. Cell viability following 1 day of culture was assayed using a CellTiter 96 Aqueous One Solution Cell Proliferation Assay kit (Promega, California, USA) and the optical density was measured at 490 nm.

### 2.6. Co-Immunoprecipitation Analysis and LC-MS/MS

First, 293T cells were transduced with recombinant lentivirus expressing JEV NS3. At 30 h post-transduction, cells were collected and lysed with NP40 lysis buffer containing protease inhibitor in ice for 30 min. Samples were subjected to centrifugation for 10 min at 4 °C to remove cellular debris. Cell lysates were incubated with anti-Flag M2 affinity gel in a rolling incubator at 4 °C overnight. Lysates were discarded after a brief centrifugation at 3000× g for 5 min at 4 °C. The beads were washed five times with cold lysis buffer prior to elution using Flag peptide. The purified products were separated by sodium dodecyl sulfate-polyacrylamide gel electrophoresis (SDS-PAGE) and visualized by silver staining. The stained bands were excised and digested. The solution was subjected to direct nanoflow liquid chromatography-tandem mass spectrometry (LC-MS/MS) analysis to identify JEV NS3 interaction proteins by Shanghai Applied Protein Technology (Shanghai, China).

When protein A/G plus-agarose (Santa Cruz, Dallas, Texas, USA) was used for co-immunoprecipitation (Co-IP), the cells lysates were first incubated with indicated antibodies at 4 °C for 5 h. Five hours later, samples were subjected to centrifugation at 3000× *g* for 1 min at 4 °C. Then the supernatant was carefully transferred into Protein-A/G plus-agarose that had been washed with lysis buffer. Samples were incubated in a rolling incubator at 4 °C for 4 h. Supernatants were discarded after a brief centrifugation at 3000× *g* for 5 min at 4 °C. The beads were washed five times with cold lysis buffer prior to elution by incubation at 95 °C in 1× sample buffer. The bound proteins were boiled in loading buffer and further analyzed by Western blotting with the appropriate antibodies.

### 2.7. Indirect Immunofluorescence

In order to observe the cellular localization of endogenous ILF2 with NS3 during JEV infection in 293T cells, 293T cells in 24-well plates were infected with JEV (5 MOI) for 24 h. The cells were washed with phosphate-buffered saline (PBS) followed by fixation with 4% ice-cold paraformaldehyde for 20 min. They were then permeabilized with PBS containing 0.1% Triton X-100 at room temperature (RT) for 10 min. The cells were washed three times with PBS, and then incubated in blocking solution (1% BSA in PBS) for 1 h. Viral and cellular proteins expression was detected with the appropriate primary antibodies for 2 h. After washing with PBS, the cells were incubated with florescence-conjugated secondary antibodies for 1 h. The cells were washed three times again with PBS before the nuclei were stained with 4’, 6’-diamidino-2-phenylindole dihydrochloride (DAPI, Sigma) for 10 min. The cells were finally washed and fluorescent images were captured with a confocal laser scanning microscope (Carl Zeiss MicroImaging, Inc., Oberkochen, Germany).

### 2.8. Western Blot Assays

Cells were harvested and treated with lysis buffer containing 1.19% HEPES, 0.88% NaCl, 0.04% EDTA, 1% NP40, and a protease inhibitor (Roche, Welwyn Garden, UK). The protein concentration of whole-cell lysates was determined with a bicinchoninic acid protein assay kit (Thermo Scientific). Equal amounts of proteins were loaded and separated using 12% sodium dodecyl sulfate polyacrylamide gels (SDS-PAGE) and transferred onto polyvinylidene fluoride (PVDF) membranes (Roche, UK). The membranes were blocked with 5% non-fat milk in 1× phosphate-buffered saline (PBS) with 5% Tween-20 (DGBio, Beijing, China) for 4 h at room temperature (RT), and subsequently incubated with diluted primary antibodies against the indicated proteins at RT or at 4 °C for 2 h or overnight. Anti-rabbit or anti-mouse IgG antibodies conjugated to horseradish peroxidase (HRP) were used as secondary antibodies and developed using an enhanced chemiluminescence substrate (Thermo Scientific, USA). All immunoblot images were performed using a Bio-Rad ChemiDoc XRS+ instrument and image software. The expression of GAPDH was detected as the internal reference with anti-GAPDH monoclonal antibodies (Abclonal).

### 2.9. Quantitative Real-Time PCR

Cells were harvested at the indicated time points for total RNA extraction. Total RNA was extracted with TRIzol reagent (Invitrogen, Grand Island, NY, USA) in accordance with the manufacturer’s instructions. Then, 1.0 μg total RNA was used to reverse transcribed into cDNA using a First-Strand cDNA Synthesis Kit (Toyobo). The mRNA levels were determined by relative quantitative real-time PCR using SYBR Green Real-Time PCR Master Mix (TOYOBO) with specific primer pairs (listed in Table 1). The relative RNA levels of specific RNA were normalized to that of the housekeeping gene GAPDH or firefly luciferase and the fold modulation in gene expression was analyzed with the 2^−ΔΔCt^ method. All reactions were performed in triplicate.

### 2.10. Plaque Assay

First, 293T cells were transduced with recombinant lentivirus carrying ILF2 or sh-ILF2 and infected with JEV at an MOI of 1.0 at 48 h after transduction. The culture supernatant was harvested at different points after infection, and the progeny virus titers were determined using a plaque-forming unit (PFU) assay. The viral culture supernatants with 10-fold dilutions were inoculated onto the monolayers of BHK-21 cells in 12-well plates. After 2 h of absorption, cells were washed with serum-free DMEM and incubated for 3 or 5 days in DMEM containing 2% FBS and 2% low melting-point agarose (GENVIEW). The cells were fixed with 10% formalin and stained with 1% crystal violet for 2 h. Visible plaques were counted and statistically analyzed; all virus titers are expressed as PFU/milliliter (PFU/mL).

### 2.11. Luciferase Reporter Assay

The plasmid carrying the JEV sub-genomic replicon fused with a Luciferase reporter gene was transcribed in vitro using the T7 MEGAscript kit (Ambion) following the manufacturer’s instructions. In brief, 293T cells were seeded in 24-well plates and transduced with sh-ILF2 expressing lentiviruses. At 36 h post-transduction, the cells were co-transfected with in vitro-transcribed JEV replicon RNA (0.5 µg) or the JEV replication-deficient replicon plasmid (0.5 µg) plus a firefly Luciferase plasmid (5 ng) as an internal control. At the indicated time points post-transfection, cell lysates were collected for the firefly and Renilla luciferase activities using a Dual-Luciferase Reporter Assay System (E1910, Promega, Madison, WI, USA).

### 2.12. Statistical Analysis

All experiments were determined in triplicate. GraphPad Prism software Version 5 (GraphPad Prism Version 5, GraphPad Software, La Jolla, CA, USA, 2012) was used in this study. The various treatments were compared by an unpaired, two-tailed Student’s *t*-test while assuming unequal variance. *p* < 0.05 was considered statistically significant. Meanwhile, *p* < 0.01 and *p* < 0.001 are marked with two (**) and three (***) asterisks, respectively.

## 3. Results

### 3.1. Identification of Host Cellular Proteins Interacting with JEV NS3

**Figure 1:**
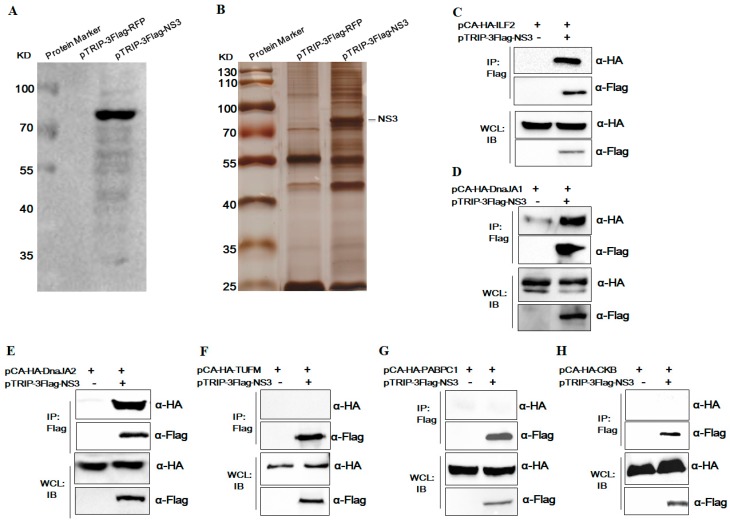
Screening of host cellular proteins interacts with Japanese encephalitis virus (JEV) NS3. Human embryonic kidney (293T) cells were transduced with recombinant lentivirus expressing JEV NS3, the immunoprecipitation with anti-Flag antibodies and LC-MS/MS analysis were successfully performed. (**A**) The expression of JEV NS3 in lentivirus-transduced 293T cells was confirmed by Western blot analysis. (**B**) Cells were collected and cell lysates were analyzed by immunoprecipitation analysis with anti-Flag antibodies. The purified proteins were separated by SDS-PAGE and visualized using silver staining. (**C**–**H**) To confirm the interaction between the candidate proteins and JEV NS3, 293T cells were co-transfected with expression plasmids encoding Flag-tagged NS3 and HA-tagged interleukin enhancer-binding factor 2 (ILF2)/DnaJA1/DnaJA2/CKB/TUFM/PABPC1, and then co-immunoprecipitation (Co-IP) was performed using anti-Flag beads and anti-HA antibodies. WCL, whole cell lysates.

To identify novel host cellular proteins interacting with JEV NS3, 293T cells were transduced with recombinant lentivirus expressing JEV NS3 and LC-MS/MS analysis was successfully performed. The expression of JEV NS3 in 293T cells was confirmed by Western blot analysis (Figure 1A). Cells were collected and cell lysates were analyzed by immunoprecipitation analysis with anti-Flag antibodies. The purified proteins were separated by SDS-PAGE and visualized using silver staining. A protein band with a molecular mass of about 72 kDa (consistent with NS3-3Flag) along with several co-purified protein bands were observed (Figure 1B). The differential protein bands were then excised and analyzed using liquid chromatography-tandem mass spectrometry (LC-MS/MS). LC-MS/MS analysis showed that several proteins (data not shown) with high hit scores such as ILF2, DnaJA1, DnaJA2, CKB, TUFM, and PABPC1 were the candidate proteins interacting with JEV NS3.

Furthermore, we confirmed the interaction between the candidate proteins and JEV NS3 through co-immunoprecipitation (Co-IP). First, 293T cells were co-transfected with expression plasmids encoding Flag-tagged NS3 and HA-tagged ILF2/DnaJA1/DnaJA2/CKB/TUFM/PABPC1, and then Co-IP was performed using anti-Flag antibodies and anti-HA antibodies. The results confirm that ILF2, DnaJA1, and DnaJA2 were co-precipitated with JEV NS3, while TUFM, PABPC1, and CKB were not (Figure 1C–F).

### 3.2. The Special Interaction of ILF2 and NS3 was not Mediated by DNA or RNA

**Figure 2:**
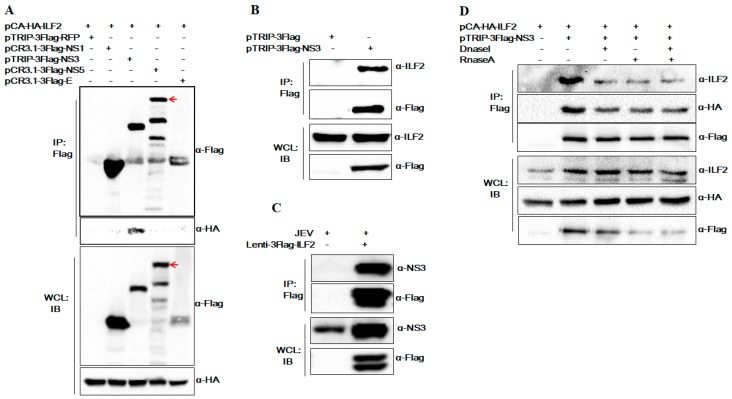
RNA or DNA are not involved in the interaction between ILF2 and JEV NS3. (**A**) The co-transfection of HA-tagged ILF2 with Flag-tagged NS3/NS5/NS1/E was conducted and cell lysates were analyzed by immunoprecipitation analysis with anti-Flag beads 24 h post-transfection. Western blot assay was then detected with the indicated antibodies. (**B**) The interaction between endogenous ILF2 and JEV NS3 was confirmed by co-precipitation. 293T cells were transfected with pTRIP-3Flag-NS3 or pTRIP-3Flag. Cell extracts were subjected to immunoprecipitation (IP) using anti-Flag beads and detected with anti-ILF2 or anti-Flag antibodies. (**C**) The interaction of ILF2 with NS3 was examined in JEV-infected 293T cells. 293T cells were transduced with lentiviruses expressing 3Flag-ILF2 for 48 h. Cells were infected with JEV at an MOI of 1.0 and cell lysates were harvested at 24 h post-infection for co-immunoprecipitation with anti-Flag beads, and were then detected with anti-NS3 polyclonal antibodies or anti-Flag antibodies. (**D**) 293T cells were co-transfected with pCA-HA-ILF2 and pTRIP-3Flag-NS3 plasmid for 24 h. Then, cell lysates treated with or without RNase A and Dnase I were harvested for co-immunoprecipitation with anti-Flag beads, and then detected with anti-HA and anti-Flag antibodies.

ILF2 is an important host cellular protein involved in the replication process of various viruses. We focused on the role of ILF2 in the infection of JEV in this study. Previous studies have reported that JEV NS3 is associated with NS5 and forms the replicase complex [8]. In order to ensure the special interaction between ILF2 and NS3, we performed co-transfection experiments to detect the interaction of ILF2 with the other JEV proteins, Flag-tagged NS3/NS5/NS1/E. The results of Co-IP and Western blot assay show that ILF2 does not co-precipitate with JEV NS5, NS1, and E proteins (Figure 2A). Then the interaction between endogenous ILF2 and JEV NS3, as well as ILF2 with JEV infection, was further confirmed by co-precipitation with Flag-tagged NS3 in 293T cells (Figure 2B,C).

ILF2 is capable of binding to nucleic acid and interacting with numerous proteins and RNAs [27,31]. To observe the DNA or RNA engaged in the interaction between ILF2 and NS3, RNaseA or Dnase I treatment prior to the co-immunoprecipitation assays was conducted. As shown in Figure 2D, the pre-treatment did not decay the interaction between ILF2 and JEV NS3, indicating that the interaction of ILF2 and NS3 was not mediated by DNA or RNA.

### 3.3. ILF2 Co-Localized with JEV NS3 during Virus Infection

**Figure 3:**
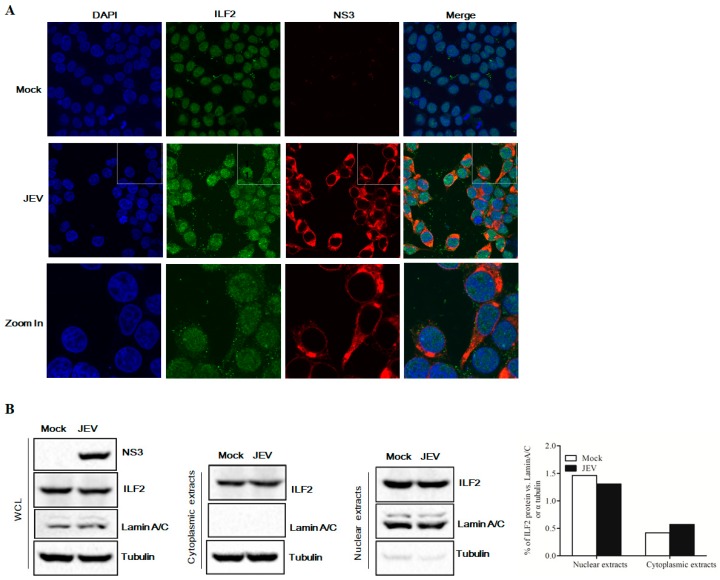
Co-localization of ILF2 with JEV NS3 in virus-infected cells. (**A**) 293T cells were infected with JEV at an MOI of 5 for 24 h, the cells were then fixed with 4% paraformaldehyde and subjected to immunofluorescence assay using anti-ILF2 antibodies (green) and anti-NS3 antibodies (red). The nuclei were stained with DAPI (blue). The images of cells were acquired using confocal microscopy (Carl Zeiss MicroImaging, Inc.). (**B**) Western blot analysis of the distribution of ILF2 in the nuclear and cytoplasmic fractions in 293T cells infected with JEV at an MOI of 1.0. Cell extracts were fractionated at 24 h post-infection (hpi), and JEV NS3 and ILF2 in the nuclear and cytoplasmic fractions were detected by immunoblotting with rabbit anti-NS3 antibodies and rabbit anti-ILF2 antibodies. LaminA/C and α-tubulin were used as nuclear and cytoplasmic markers, respectively.

To verify the intracellular co-localization of ILF2 with NS3 in JEV-infected cells, 293T cells were infected with JEV at an MOI of 5, and confocal immunofluorescence assay was performed 24 h post-infection (hpi). As shown in Figure 3A, ILF2 was detected mainly in the nucleus in mock-infected cells; however, ILF2 partly translocated from the nucleus to cytoplasm and co-located with NS3 in JEV-infected cells. Moreover, to examine the subcellular localization of ILF2, the cytoplasmic and nuclear fractions of JEV-infected 293T cells (1.0 MOI) were separated. The cell fractionation was analyzed by immunoblotting and the results show that ILF2 was detected in both the nuclear and cytoplasmic fractions, while the amount of ILF2 was increased in the cytoplasm and decreased in the nucleus in JEV-infected cells (Figure 3B).

### 3.4. Expression of ILF2 in 293T Cells Infected with JEV

**Figure 4:**
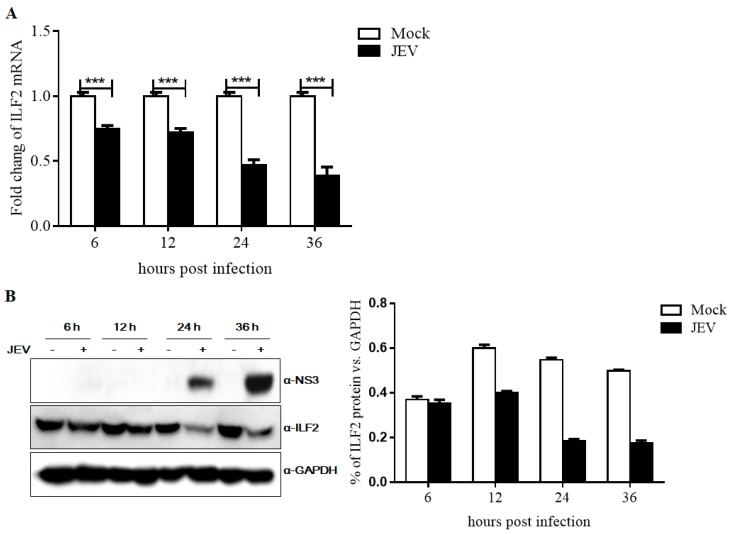
Expression of ILF2 in 293T cells infected with JEV. (**A**) 293T cells were uninfected or infected with JEV at an MOI of 1.0. At different times post-infection, samples were collected and the ILF2 mRNA level was determined by quantitative real-time PCR and normalized to GAPDH. (**B**) Protein levels of ILF2 were evaluated through Western blot analysis and GAPDH was used as a normalizer (left). The relative protein levels were analyzed using Image J software (right). The data shown are from three independent assays, with the error bars representing the standard deviations (*** *p* < 0.001, Student’s *t*-test).

To assess the response of ILF2 to JEV infection, 293T cells were infected with JEV at an MOI of 1.0 and collected at indicated time points, and ILF2 mRNA and protein levels of ILF2 were evaluated by qRT-PCR and Western blotting analysis. As shown in Figure 4A, the mRNA level of ILF2 was decreased at 6 h to 36 h after JEV infection compared with the control group in 293T cells. The protein level of ILF2 significantly decreased at 24 h and 36 h post-infection with JEV, which was correlated with the expression of JEV NS3 (Figure 4B). These data clearly indicate that JEV infection significantly decreased the expression of ILF2 at both the mRNA and protein levels.

### 3.5. ILF2 Inhibits JEV Replication

**Figure 5:**
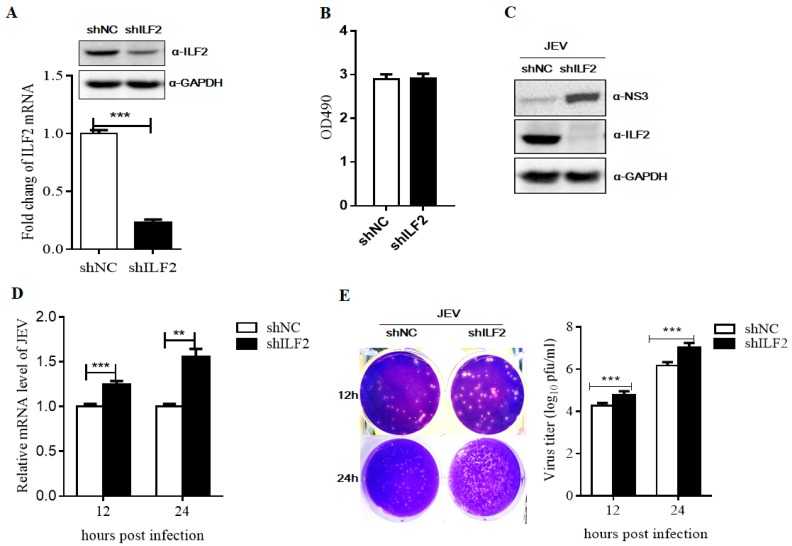
Knockdown of ILF2 promotes JEV infection. (**A**–**B**) 293T cells were transduced with lentivirus short hairpin RNA (shRNA) targeting ILF2 (shILF2) or non-target shRNA (NC) for 48 h. The silence efficiency was measured by Western blot and real-time PCR analysis (A) and the proliferation of 293T-shILF2 cells was analyzed by MTS assay (B). 293T-shILF2 and 293T-shNC cells were infected with JEV at an MOI of 1.0 for 12 h or 24 h. Cells supernatants and total RNA were harvested for indicated experiments. (**C**) The protein level of JEV NS3 was conducted through Western blot analysis with anti-NS3 antibodies and normalized to GAPDH. (**D**) The relative quantification of intracellular JEV C gene copies was detected by real-time PCR analysis. (**E**) The production of progeny virus titer in culture supernatants was measured by plaque assay in baby hamster kidney (BHK)-21 cells. The data shown are from three independent assays, with the error bars representing standard deviations (* *p* < 0.05; ** *p* < 0.01; *** *p* < 0.001, Student’s *t*-test).

To explore the role of ILF2 in JEV replication, 293T cells were transduced with recombinant lentiviruses carrying shRNA corresponding to the ILF2 mRNA sequences (shILF2) or control lentiviruses (shNC, Negative Control (NC)). The interference effect was detected by Western blot and real-time PCR. The results show that protein and mRNA levels of ILF2 were significantly decreased compared to the control cells (Figure 5A). MTS assays demonstrated that the knockdown of ILF2 does not affect 293T cell proliferation (Figure 5B). The control and ILF2-knockdown 293T cells were both infected with JEV at an MOI of 1. The culture supernatants were harvested; the NS3 protein level of JEV and gene copies of the JEV *C* gene were detected by Western blot and qRT-PCR analysis. As shown in Figure 5C,D, the NS3 protein levels were significantly increased in the knockdown of ILF2 compared with the control cells. The *C* gene levels of JEV were also significantly increased in the ILF2-knockdown 293T cells. Moreover, the titers of the progeny virus were determined using a plaque-forming unit assay, and the results show that the knockdown of ILF2 significantly increased virus titers (Figure 5E).

**Figure 6:**
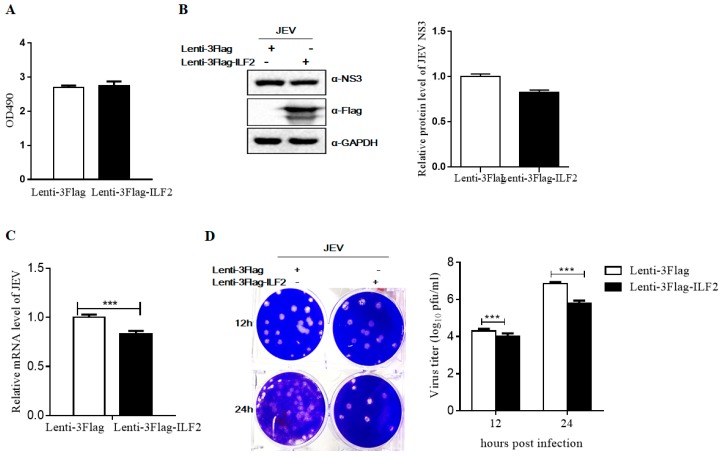
Overexpression of ILF2 inhibits JEV replication in 293T cells. 293T cells were transduced with lentiviruses expressing ILF2 (Lenti-3Flag-ILF2) or a control lentivirus (Lenti-3Flag) for 48 h and the proliferation of overexpressing ILF2 293T cells was analyzed by MTS assay. (**A**) Cells were infected with JEV at an MOI of 1.0 for 12 h or 24 h. Cells supernatants and total RNA were harvested for the indicated experiments. The antiviral activity of ILF2 against JEV infection was evaluated with different experiments. (**B**) Western blot analysis of the protein level of JEV NS3 was performed using the indicated antibodies. GAPDH served as the loading control at an equal sample loading. (**C**) The relative quantification of intracellular JEV C gene copies by real-time PCR analysis. (**D**) The production of progeny virus in culture supernatants was measured by plaque assay. The plaque formation ability was observed in BHK-21cells (left) and virus titer was calculated (right). Three asterisks indicate that a statistical difference exists between overexpressing ILF2 293T cells and control cells at *p* < 0.001.

We further investigated whether ectopic expression of ILF2 could inhibit JEV replication in 293T cells. Cells were transduced with recombinant lentiviruses expressing ILF2 (Lenti-3Flag-ILF2) and a control (Lenti-3Flag). MTS assay showed that the overexpression of ILF2 does not affect cell proliferation (Figure 6A). As expected, JEV replication was significantly inhibited at different time points post-infection in 293T cells overexpressing ILF2, determined by Western blot, qRT-PCR, and PFU assays (Figure 6B–D). All these results indicate that ILF2 is a host restriction factor for JEV.

### 3.6. Knockdown of ILF2 Positively Regulates JEV Replication by Sub-Genomic Replicon System Assay

**Figure 7:**
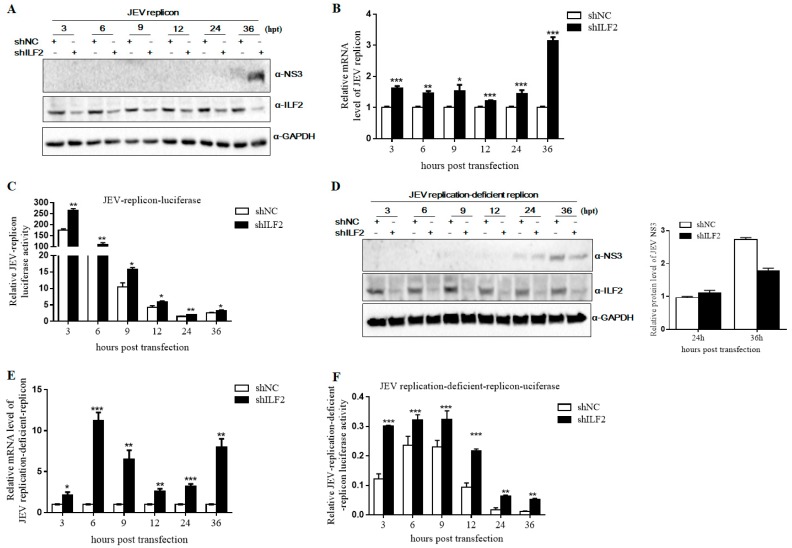
Knockdown of ILF2 positively regulates JEV replication by sub-genomic replicon system assay. 293T-shILF2 cells and 293T-shNC cells were transfected with a Renilla luciferase JEV replicon RNA or a Renilla luciferase JEV replication-deficient replicon DNA. Firefly luciferase was used as the internal control. Cells lysates were harvested at 3, 6, 9, 12, 24, and 36 h post-transfection (hpt). (**A**,**D**) The protein level of JEV NS3 was determined by Western blot analysis with antibodies against JEV NS3, ILF2, and GAPDH. GAPDH served as the loading control at an equal sample loading. (**B**,**E**) Total cellular RNA was extracted and the level of JEV RNA (NS3) was detected through real-time PCR analysis. (**C**,**F**) Cell lysates were collected at the indicated times and were subjected to dual-luciferase assay. Renilla luciferase activity was normalized to firefly luciferase. The data shown are from three independent assays, with the error bars representing standard deviations (* *p* < 0.05; ** *p* < 0.01; *** *p* < 0.001, Student’s *t*-test).

Our previous study showed that part of ILF2 was translocated from the nucleus to cytoplasm and co-located with NS3 in JEV-infected cells. We hypothesized that ILF2 might be involved in viral translation and genome replication to inhibit JEV replication. Then, we used a sub-genomic replicon system to measure viral replication activity. ILF2-knockdown 293T cells by special shRNA were transfected with in vitro-transcribed JEV replicon RNA and firefly luciferase DNA was used as the internal control. Cells were harvested at 3, 6, 9, 12, 24, and 36 h post-transfection (hpt). Western blot analysis showed that the level of NS3 protein was extremely increased in ILF2-knockdown 293T cells compared with the control cells at 36 hpt (Figure 7A). qRT-PCR assay showed that the replicon RNA was also markedly increased in ILF2-knockdown 293T cells compared with the control cells at 3, 6, 9, 12, 24, and 36 hpt (Figure 7B). The activity of luciferase in the replicon was measured by dual luciferase assays. As shown in Figure 7C, the luciferase activity was higher for all of the time points in ILF2-knockdown cells compared with that of the control cells. Our results suggest that ILF2 may participate in viral protein translation and/or virus RNA replication.

JEV RNA replication depends on viral proteins, and JEV NS3 and NS5 proteins have been shown to play important roles in viral genome replication [6,37]. We further used a replication-deficient replicon containing a GDD-AAG mutation in NS5 to study the effect of ILF2 on viral translation. The ILF2-knockdown 293T cells were transfected with JEV replication-deficient replicon plasmid and firefly luciferase DNA as above. Western blot analysis and the quantification of the JEV NS3 band’s intensity were analyzed by ImageJ software and the results show that the protein products of JEV NS3 were increased compared with the control cells at 24 hpt, while they were decreased at 36 hpt (Figure 7D). The replicon RNA and luciferase activities were higher in the ILF2-knockdown 293T cells (Figure 7E,F). These data demonstrate that the knockdown of ILF2 favored JEV genome replication.

## 4. Discussion

Host antiviral restriction factors, including interferon-stimulated genes (ISGs) and other antiviral factors, can target various steps in the virus life cycle. The interaction of virus and host factors is a key step for the virus infection process and plays an important role in viral RNA replication, translation, and post-translational modification, aiding in our understanding of the molecular mechanism of viral pathogenesis and the development of antiviral drugs [38]. In recent years, several studies have identified anti-JEV host restriction factors with different mechanisms [17,39,40,41,42,43,44,45]. In this study, host cellular protein ILF2 was identified to specifically interact with JEV NS3. We demonstrate that the overexpression of ILF2 inhibits JEV infection and the knockdown of endogenous ILF2 enhances JEV replication. Furthermore, the knockdown of ILF2 favors JEV genome replication by positively regulating JEV protein translation.

JEV NS3 is a multifunctional protein that functions as helicase, protease, and nucleoside triphosphatase by interacting with JEV NS2B and NS4A. Moreover, NS3 forms the viral replicase complex together with NS5 and dsRNA [4,8] and plays an important role in viral replication [12,46] and viral assembly [36]. In this study, IP combined with LC-MS/MS assays were performed to further explore new host proteins interacting with JEV NS3. It was demonstrated that JEV NS3 specifically interacts with cellular ILF2 (Figure 1). ILF2 was originally identified to regulate interleukin-2 gene transcription via interaction with the antigen receptor response element and modulate interleukin-2 (IL-2) promoter as a sequence-specific DNA-binding protein. ILF2 was also considered as an important regulatory factor involved in the replication process of various types of viruses.

We further confirmed the interaction of ILF2 with JEV NS3 by co-immunoprecipitation (Co-IP), Western blotting, and immunofluorescence assay. ILF2 did not co-precipitate with NS5 through co-transfection experiments of ILF2 with NS5 in our study (Figure 2C). Meanwhile, the interaction between JEV NS3 and ILF2 was not mediated by RNA and DNA. ILF2 is abundant in many cell types and tissues, being primarily distributed in the nuclear fractions but also present in the cytoplasm of many different cell types [23,47]. The subcellular localization of cellular proteins can highlight their important biological functions. We found that cellular ILF2 could be translocated partly from the nucleus to the cytoplasm and co-localized with the NS3 of JEV in the cytoplasm of JEV-infected 293T cells, while ILF2 was mostly localized in the nucleus of mock-infected 293T cells. This finding is consistent with the previous reports that ILF2 could be translocated partly from the nucleus to the cytoplasm and co-localized with virus proteins in IBDV-, HCV-, and PRRSV-infected cells [27,28,33]. Moreover, the expression of ILF2 was significantly reduced after JEV infection. All data indicate that ILF2 may play a critical role in JEV replication.

To explore the antiviral potential of ILF2 against JEV, we analyzed the effect of ILF2 knockdown by shRNA and ILF2 overexpression through lentiviral transduction. It was demonstrated that the downregulation of endogenous ILF2 enhanced JEV replication and the overexpression of ILF2 inhibited JEV infection (Figure 5 and Figure 6). The antiviral properties of ILF2 with different strategies have been reported. ILF2 participated in the regulation of BVDV translation and replication via binding to 3’NTR (Nontranslated region, NTR) of the BVDV genome [48]. It was also reported that ILF2 could specifically bind to replication signals in the HCV genomic RNA and was involved in HCV RNA replication [27]. In this report, we found that ILF2 was not involved in the entry process of JEV (data not shown). Furthermore, the JEV sub-genomic replicon system assay showed that a higher mRNA level of JEV replication-deficient replicon was observed in 293T-shILF2 as compared to 293T-shNC cells (Figure 7), which could lead to a higher level of luciferase activities and NS3 protein at 24 hpt. The detailed mechanism remains uncertain in this study. 

## 5. Conclusions

In conclusion, we demonstrated that cellular ILF2 possesses antiviral activity against JEV in vitro. Thus, our study indicated that ILF2 exerted antiviral activity against JEV infection and could provide valuable protein targets for antiviral strategies in the future. However, further studies are warranted to more precisely elucidate the molecular mechanisms, such as the expression of ILF2 after JEV infection as well as the antiviral action of ILF2 against JEV in vivo.

## Figures and Tables

**Table 1:** List of primers used in this study.

Primers	Oligonucleotide Sequence (5′–3′)
pCAGGS-HA-ILF2-F	GAATTCATGAGGGGTGACAGAGGC
pCAGGS-HA-ILF2-R	CTCGAGCTCCTGAGTTTCCATGC
pTRIP-ILF2-3Flag-F	ACTAGTATGAGGGGTGACAGAGGCCGTGG
pTRIP-ILF2-3Flag-R	GTCGACCTCCTGAGTTTCCATGCTTTC
pCAGGS-HA-Firefly-F	GAATTCATGGAAGATGCCAAAAACATTAAG
pCAGGS-HA-Firefly-R	CTCGAGCACGGCGATCTTGCCGCCCTTC
qILF2-F	AGTCGTGGAAAGCCTAAGAG
qILF2-R	GGTGGCACTGTTGTAATGAG
qFirefiy-F	GGCCCCAGCTAACGACATCTAC
qFirefly-R	GCAGCCCTTTCTTGCTCACG
qGAPDH-F	CACCCACTCCTCCACCTTTG
qGAPDH-R	CCACCACCCTGTTGCTGTAG
qJEV-C-F	GAGCTTGTTGGACGGCAGAG
qJEV-C-R	CACGGCGTCGATGAGTGTTC

p: primer sequence for the construction of expression plasmids; q: primer sequence for quantitative real-time PCR analysis; F: forward; R: reverse.
